# Six new species of the genus *Exocelina* Broun, 1886 from Wano Land, New Guinea (Coleoptera, Dytiscidae, Copelatinae)

**DOI:** 10.3897/zookeys.665.11792

**Published:** 2017-04-04

**Authors:** Helena Shaverdo, Michael Wild, Bob Sumoked, Michael Balke

**Affiliations:** 1 Naturhistorisches Museum Wien, Burgring 7, 1010 Vienna, Austria; 2 PO Box 369, Sentani 99352, Jayapura, Papua, Indonesia; 3 Walian 2, Tomohon Selatan, N Sulawesi 95439, Indonesia; 4 SNSB-Zoologische Staatssammlung München, Münchhausenstraße 21, D-81247 Munich, Germany and GeoBioCenter, Ludwig-Maximilians-University, Munich, Germany

**Keywords:** Copelatinae, Dytiscidae, *Exocelina*, New Guinea, new species, key

## Abstract

Six new species of New Guinea *Exocelina* Broun, 1886 are described in this paper: *E. iratoi* **sp. n.**, *E. likui* **sp. n.**, *E. pui* **sp. n.**, *E. pulukensis* **sp. n.**, *E. tomhansi* **sp. n.**, and *E. wigodukensis* **sp. n**. Although different morphologically, together with *Exocelina ascendens* (Balke, 1998), *E. bagus* (Balke & Hendrich, 2001), and *E. ransikiensis* Shaverdo, Panjaitan & Balke, 2016, they are found to form a monophyletic clade and be closely related to representatives of the *E. ekari*-group, based on preliminary analysis of sequence data. An identification key to the species is provided, and important diagnostic characters are illustrated. The present data on the species’ distribution show that most of them are local endemics.

## Introduction

This paper is in continuity with our previous taxonomic studies on the New Guinea species of the diving beetle genus *Exocelina* Broun, 1886 (Balke 1998, 1999, Shaverdo and Balke 2014, Shaverdo et al. 2005, 2012, 2013, 2014, 2016a, b, c, d). *Exocelina* species have also been used as a model to reveal lineage diversification trends in New Guinea where the complex geological formation of the island gave rise to intriguing biogeographic patterns (Toussaint et al. 2014). The genus also served as a model taxon to study diversification across major Melanesian islands (Toussaint et al. 2015).

**Figure 1.**
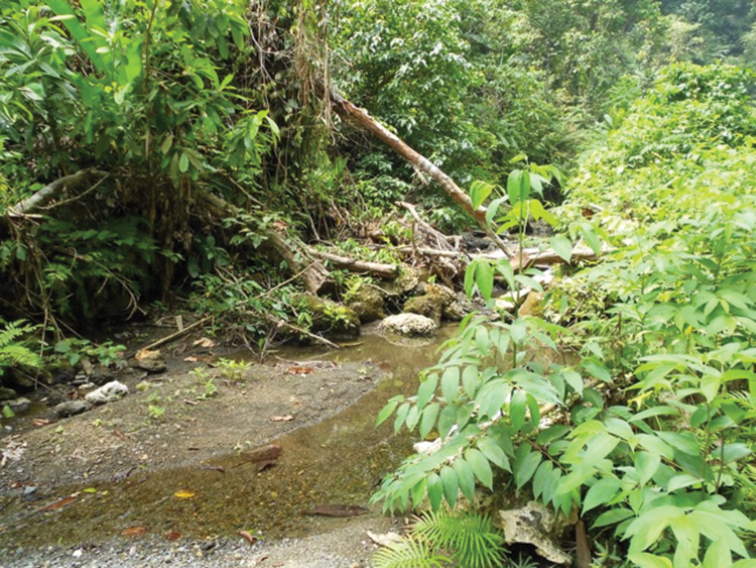
Wano Land, Mokndoma, small forest stream; photo by M. Wild.

**Figure 2.**
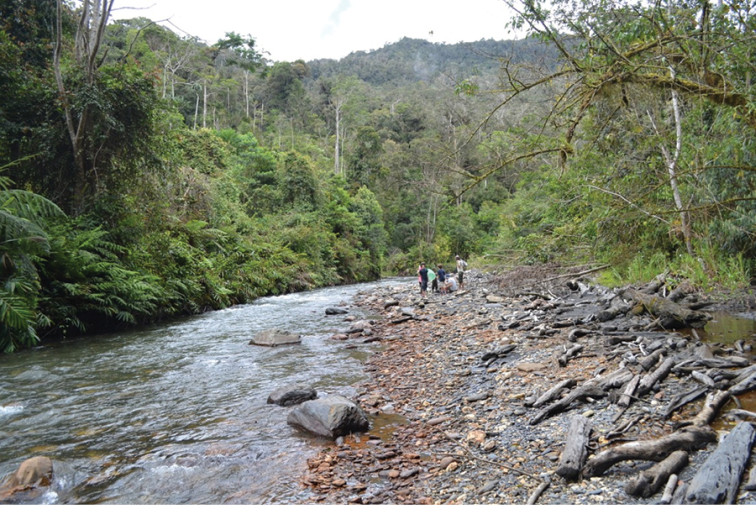
Wano Land, Wigoduk, Nggoduk River; photo by M. Wild.

Here, the discovery of six new *Exocelina* species is reported as the result of surveys in the most remote heart of Papua, at the interface of the central highlands and the central Papuan lake plains. The preliminary molecular analysis of these species and old material of *Exocelina ascendens* (Balke, 1998) from the Star Mountains of the eastern Papua, suggests that they form a monophyletic clade which also includes *E. bagus* (Balke & Hendrich, 2001) and the recently described *E. ransikiensis* Shaverdo, Panjaitan & Balke, 2016. As shown on the example of *E. bagus* and *E. ransikiensis* (undescribed sp. MB1269) in the phylogenetic trees (Figs 1–2) by Toussaint et al. (2014), this clade is closely related to the representatives of the *E. ekari*-group and consists of very distinct, morphologically isolated lineages, which we treat as species groups here. An identification key to all included species is provided.

## Materials and methods

The present work is based on the material from the following collections:

**CGW** collection of Dr. Günther Wewalka, Vienna, Austria

**MZB** Museum Zoologicum Bogoriense, Cibinong, Indonesia

**NHMW** Naturhistorisches Museum Wien, Vienna, Austria

**ZSM** Zoologische Staatsammlung München, Munich, Germany

All methods follow those described in detail in our previous articles (Shaverdo and Balke 2014, Shaverdo et al. 2012, 2014).

## Checklist and distribution of the species

### Species descriptions

**Table:** Abbreviations: P – Papua; WP – West Papua.

	***Exocelina ascendens*-group**	
1.	*Exocelina ascendens* (Balke, 1998)	P: Pegunungan Bintang
2.	*Exocelina tomhansi* sp. n.	P: Puncak Jaya, Puncak
	***Exocelina bagus*-group**	
3.	*Exocelina bagus* (Balke & Hendrich, 2001)	P: Nabire
	***Exocelina iratoi*-group**	
4.	*Exocelina iratoi* sp. n.	P: Puncak
	***Exocelina likui*-group**	
5.	*Exocelina likui* sp. n.	P: Puncak Jaya
	***Exocelina pui*-group**	
6.	*Exocelina pui* sp. n.	P: Puncak
	***Exocelina ransikiensis*-group**	
7.	*Exocelina ransikiensis* Shaverdo, Panjaitan & Balke, 2016	WP: Manokwari; P: Nabire
	***Exocelina wigodukensis*-group**	
8.	*Exocelina wigodukensis* sp. n.	P: Puncak Jaya
9.	*Exocelina pulukensis* sp. n.	P: Puncak Jaya

#### *Exocelina ascendens*-group

##### 1. Exocelina ascendens

Taxon classificationAnimaliaColeopteraDytiscidae

(Balke, 1998)

Figs 3 , 11

Copelatus (Papuadytes) ascendens Balke, 1998: 322; Nilsson 2001: 76 (catalogue).
Papuadytes ascendens (Balke, 1998): Nilsson and Fery 2006: 56 (comb. n.).
Exocelina ascendens (Balke, 1998): Nilsson 2007: 33 (comb. n.).

###### Type locality.

Papua: Pegunungan Bintang Regency, trek between Aipomek and Diruemna, 04°25'S; 139°57'E, 2600 m a.s.l.

###### Type material studied.

*Holotype*: male “IRIAN JAYA, 3.9.1992 Aipomek - Diuremna [sic!] 139°57'E 04°25'S 2600m, leg.Balke (35)”, “HOLOTYPUS” [red], “Copelatus ascendens Balke des. 1997” [red] (NHMW). *Paratypes*: 4 males, 2 females with the same label as the holotype and additionally with red labels “Paratypus Copelatus ascendens Balke des. 1997”, one of the males with an additional label “M.Balke 3282” [green], another male with two additional labels “M.Balke 3283” [green] and “M.Balke 6409” [green text] (NHMW). 1 male, 2 females “IRIAN JAYA, 9.9.1992 Kono - Angguruk 139°47'E 04°19'S 2600m, leg.Balke (44)”, “Paratypus Copelatus ascendens Balke des. 1997” [red] (NHMW), note: the original description says “2 males and 1 female”, it is probably a mistake in the sex identification or type error. 4 males, 3 females “IRIAN JAYA, 24.-26.9.1993 Eipomek [sic!] Gebiet Eipomek [sic!] - Diruemna”, “ca. 140°01'E 04°27'S 1800-2600m, leg. M. Balke (21 [crossed out] -22)”, “Paratypus Copelatus ascendens Balke des. 1997” [red] (NHMW, CGW).

###### Diagnosis.

**Figures 3–6.**
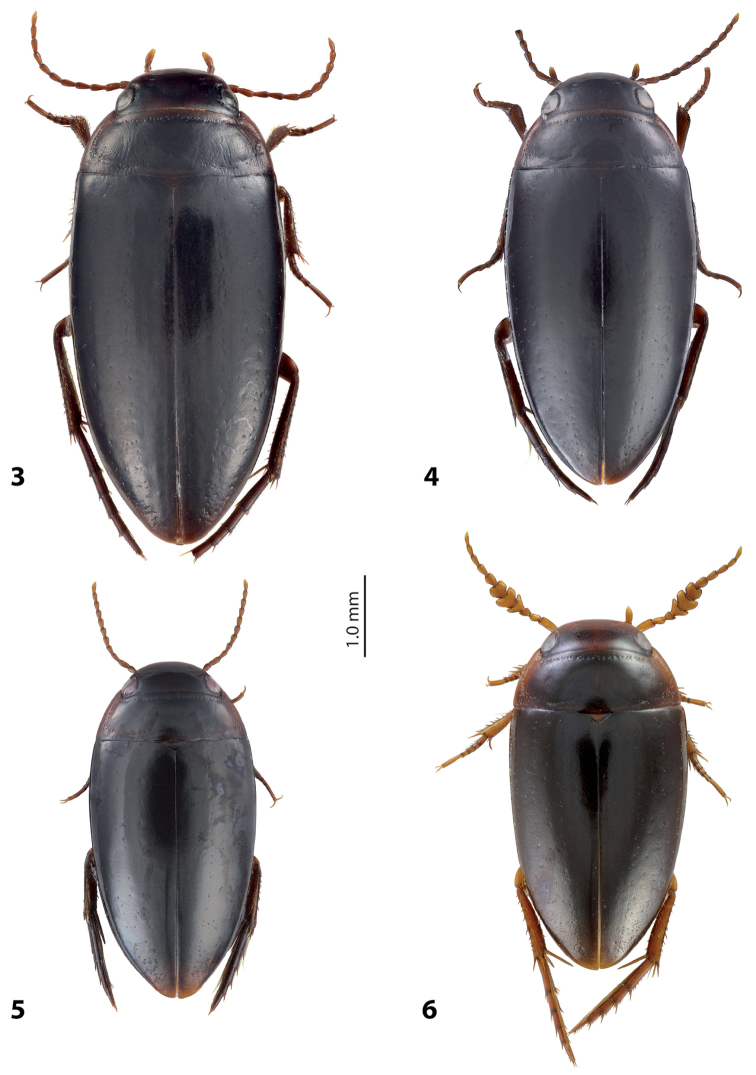
Habitus and coloration **3** *Exocelina ascendens* (Balke, 1998) **4** *E. tomhansi* sp. n. **5** *E. iratoi* sp. n. **6** *E. bagus* (Balke & Hendrich, 2001).

Beetle large (TL-H 5.3–5.75 mm), elongate; piceous, with dark brown pronotal sites and head anteriorly; submatt, with fine but evident punctation and rather strongly impressed microreticulation; pronotum with distinct lateral bead; male antennae simple, slender (Fig. 3); male protarsomere 4 with small (slightly larger than more laterally situated large seta), weakly curved anterolateral hook-like seta; male protarsomere 5 ventrally with anterior row of 23 and posterior row of 8 short, strong, spine-like setae (Fig. 11D); median lobe evenly curved, pointed in lateral view and evenly tapering, with broadly pointed apex in ventral view, on both lateral sides with numerous fine setae situated linearly on anterior half of distal part of median lobe; paramere robust, with notch on dorsal side and very dense, strong setae on subdistal part; proximal setae sparse and fine (Fig. 11A–C). For complete description, see Balke (1998).

###### Distribution.

Papua: Pegunungan Bintang Regency. The species is known only from the type material (Fig. 19).

##### 2. Exocelina tomhansi sp. n.

Taxon classificationAnimaliaColeopteraDytiscidae

http://zoobank.org/C135B374-683A-469E-BB57-F4A115245841

Figs 4 , 12

###### Type locality.

Papua: Puncak Jaya Regency, 03°36'42.5"S; 137°31'40.1"E.

###### Type material.

*Holotype*: male “Indonesia: Papua, Wano Land, S of pass to lake plains, 1700m, 2.ix.2014, -3,6117913 137,5277983, Balke & Wild (Pap022)”, “M.Balke 6512” [green text] (MZB). *Paratypes*: 1 female with the same label as the holotype and with an additional label “M.Balke 6513” [green text] (ZSM). 1 female “Indonesia: Papua, Wano Land, creek @ jungle helipad, 870m, 4.ix.2014, -3,584077 137,5042947, Balke & Wild (Pap027)” (ZSM).

###### Diagnosis.

Beetle medium-sized, oblong-oval, piceous, with brown sides of pronotum, dorsal punctation inconspicuous, microreticulation weakly impressed; pronotum without lateral bead; male antennae simple; male protarsomere 4 with large, thick, strongly curved anterolateral hook-like seta; median lobe evenly curved, pointed in lateral view and evenly tapering, with broadly pointed apex in ventral view, on both lateral sides with numerous short, thick setae situated on anterior half of distal part of median lobe; paramere robust, with notch on dorsal side and very dense, strong setae on subdistal part; proximal setae sparse and fine. The new species is very similar to *E. ascendens* in shape of the median lobe and in shape and setation of the paramere but distinctly differs from it having smaller body size, shiny dorsal surface due to much more weakly impressed microreticulation, larger anterolateral hook-like seta on the male protarsomere 4, longer setae of the male protarsomere 5 and, especially in absence of the pronotal bead.

###### Description.

*Size and shape*: Beetle medium-sized (TL-H 4.5–4.75 mm, TL 4.8–5.1 mm, MW 2.35–2.45 mm), with oblong-oval habitus, broadest at elytral middle. *Coloration*: Dorsally piceous, with reddish brown to brown narrow anterior margin of head and sides of pronotum; one female with head paler; head appendages and legs yellowish red to reddish brown, metathoracic legs darker distally (Fig. 4).

*Surface sculpture*: Head with sparse punctation (spaces between punctures 2–3 times size of punctures), evidently finer and sparser anteriorly; diameter of punctures smaller than to almost equal to diameter of cells of microreticulation. Pronotum with much sparser and finer punctation than head. Elytra with extremely sparse and fine punctation, almost invisible. Pronotum and elytra with slightly impressed microreticulation, dorsal surface shiny. Head with microreticulation stronger. Metaventrite and metacoxae distinctly microreticulate, metacoxal plates with longitudinal strioles and transverse wrinkles. Abdominal ventrites with distinct microreticulation, strioles, and very fine and sparse punctation.

*Structures*: Pronotum without lateral bead. Base of prosternum and neck of prosternal process with distinct ridge, smooth and slightly rounded anteriorly. Blade of prosternal process lanceolate, narrow, slightly convex, with distinct lateral bead and few setae; neck and blade of prosternal process evenly jointed. Abdominal ventrite 6 broadly rounded or slightly truncate apically.

*Male*: Antenna simple (Fig. 4). Pro- and mesotarsomeres 1–3 not dilated. Protarsomere 4 cylindrical, narrow, with large, thick, strongly curved anterolateral hook-like seta. Protarsomere 5 ventrally with anterior band of 26 and posterior row of six relatively long, not pointed setae (Fig. 12D). Median lobe evenly curved, pointed in lateral view and evenly tapering, with broadly pointed apex in ventral view, on both lateral sides with numerous short, thick setae situated on anterior half of distal part of median lobe. Paramere robust, with notch on dorsal side; subdistal part large and elongate, with very dense, strong setae; proximal setae sparse and fine (Fig. 12A–C). Abdominal ventrite 6 slightly truncate ventrally, with 18–19 fine lateral strioles on each side.

*Holotype*: TL-H 4.75 mm, TL 5.1 mm, MW 2.45 mm.

*Female*: Pro- and mesotarsi not modified. Abdominal ventrite 6 without lateral strioles.

###### Distribution.

Papua: Puncak Jaya and Puncak Regencies (Fig. 19).

###### Etymology.

The species is named in honour of helicopter pilot Tom Hans who has served the Papuan people for many years. The name is a noun in the genitive case.

#### *Exocelina bagus*-group

##### 3. Exocelina bagus

Taxon classificationAnimaliaColeopteraDytiscidae

(Balke & Hendrich, 2001)

Figs 6 , 14

Copelatus (Papuadytes) speciosus Balke & Hendrich, 1998: 336; Nilsson 2001: 77 (catalogue).
Copelatus (Papuadytes) bagus Balke & Hendrich, in Balke 2001: 361.
Exocelina bagus (Balke & Hendrich, 2001): Nilsson 2007: 33 (as E. baga, comb.n.).
Exocelina bagus MB4915: Toussaint et al. 2014: Supplementary figs 1–4, Tab. 2.

###### Type locality.

Papua: Nabire Regency, 54–55 km of road Nabire to Enarotali, ca. 03°29.80'S; 135°43.89'E. *Note*: the road only goes up to Enarotali, Ilaga is much further in the mountains, therefore, people now refer to the road as Nabire-Enarotali.

###### Type material studied.

*Holotype*: male “IR90-11: W. New Guinea, Trek Nabire-Ilaga, km55, 19.-25.ix.1990, Balke”, “HOLOTYPUS” [red], “Copelatus speciosus sp.n. Balke des. 1997” [red] (NHMW). *Paratypes*: 9 males, 12 females with the same label as the holotype (NHMW). 6 females “W.-Neuguinea/Paniai Prov. Strasse Nabire-Ilaga km 54 700m, 22.–25.9.1990/IR 11 leg: Balke & Hendrich” (NHMW). 7 males, 8 females “IR 20-W. New Guinea, track Nabire-Ilaga KM 59, ca.750m, 18.vii.1991, Balke & Hendrich leg.” (NHMW). 1 female “IR 21-W. New Guinea track Nabire-Ilaga KM 65, Kali Utowa, 250 M, 18–19.vii.1991 Balke & Hendrich leg.” (NHMW), note: the original description says “one male”, it is probably a mistake in the sex identification or type error. 6 females “IR 23-W. New Guinea, track Nabire-Ilaga, KM 62, 250m, 24.vii.1991 Balke & Hendrich leg.” (NHMW). 1 female “IR 24-W. New Guinea, track Nabire-Ilaga Km 54, basecamp, 750m, 25.vii.1991 Balke & Hendrich leg.” (NHMW). All paratypes are additionally with red labels “Paratypus Copelatus speciosus Balke des. 1997”.

###### Additional material.

10 males, 15 females “IR #91-7 (IR 24). West New Guinea, Nabire-Ilaga km 54, 750m, 25.&27.1991 Balke” (NHMW). 8 males, 8 females “IRIAN JAYA: Paniai Prov. road Nabire-Ilaga, km 54 26./27.8.1996, 750-800m leg. M. Balke (96 # 2)” (NHMW). 6 males, 5 females “IRIAN JAYA: Paniai Prov. road Nabire-Ilaga, km 54 30.8.1996, 750m leg. M. Balke (96 # 9)” (NHMW). 2 males “IRIAN JAYA: Paniai Prov. road Nabire-Ilaga, km 54 10.9.1996, 900m leg. M. Balke (96 # 19)” (NHMW). 7 males, 3 females “IRIAN JAYA: Paniai Prov. road Nabire-Ilaga, km 54 10.9.1996, 800m leg. M. Balke (96 # 20)” (NHMW). *Note*: although most of these mentioned above specimens are with the paratype labels, they are not included in the type material of the original description in Balke (1998, p. 336). 1 female “Indonesia: Papua, Road Nabire-Enarotali KM 55, 774m, 22.x.2011, 03 29.796S 135 43.885E, Uncen (PAP09)” (NHMW). 2 males, 1 female “Indonesia: Papua, Road Nabire-Enarotali KM 60, 640m, 22.x.2011, 03 30.474S 135 42.611E, Uncen (PAP10)” (NHMW).

###### Diagnosis.

Beetle medium-sized (TL-H 3.8–4.8 mm), elongate; dark brown, with reddish brown pronotal sites and head anteriorly; submatt, with fine but evident punctation and rather strongly impressed microreticulation; pronotum without lateral bead (Fig. 6; fig. 3 in Balke (1998)); male antennae strongly and modified: antennomeres 2–3 very strongly reduced, 4–6 excessively enlarged and 3 and 7 strongly enlarged (Fig. 6; fig. 16 in Balke (1998)); male protarsomere 4 with large, strongly curved anterolateral hook-like setae; male protarsomere 5 slightly concave ventrally, with anterior band of more than 30 and posterior row of five relatively long, not pointed setae (Fig. 14D); median lobe strongly curved, evenly tapering to apex, apex straight, pointed, with short, thick lateral setae in lateral view, subdistal part of median lobe strongly broadened in ventral view; paramere with distinct dorsal notch and large, elongate subdistal part; subdistal setae very dense, strong, long, proximal setae very sparse, thin, small, weakly visible (Fig. 14A–C; figs 39, 86, 92 in Balke (1998)). For complete description, see Balke (1998).

###### Distribution.

Papua: Nabire Regency. The species is known only from the Mount Gamey area (Fig. 19).

#### *Exocelina iratoi*-group

##### 4. Exocelina iratoi sp. n.

Taxon classificationAnimaliaColeopteraDytiscidae

http://zoobank.org/CAB3EE36-F390-425C-8BBE-7C1BE87B582D

Figs 5 , 13

###### Type locality.

Papua: Puncak Regency, south from Iratoi, 03°54'20.4"S; 137°12'03.2"E.

###### Type material.

*Holotype*: male “Indonesia: Papua Province, S Iratoi, forest, 378m, 22.v.2015, -3,3904028031975, 137,3201, Sumoked & Balke (Pap037)”, “M.Balke 6984” [green text] (MZB). *Paratypes*: 1 male with the same label as the holotype (NHMW). 2 females “Indonesia: Papua Province, S Iratoi, forest, 553m, 22.v.2015, -3,391922694, 137,3235278, Sumoked & Balke (Pap038)”, one of them with an additional label “M.Balke 6985” [green text] (ZSM).

###### Diagnosis.

Beetle small, oblong-oval, piceous, with dark brown head and pronotum, dorsal punctation inconspicuous, microreticulation weakly impressed; pronotum without lateral bead; male antennae simple; male protarsomere 4 with large, thick, strongly curved anterolateral hook-like seta; apex of median lobe with three small prolongations; paramere without dorsal notch, with long, rather dense, thin setae, situated along dorsal margin, not clearly divided into subdistal and proximal.

In oblong-oval shape of the body, fine dorsal sculpture, and absence of the pronotal bead, the species is similar to many small species of the *E. ekari*-group but distinctly differs from them in shape of the median lobe and paramere.

###### Description.

*Size and shape*: Beetle small (TL-H 3.7–3.9 mm, TL 4–4.25 mm, MW 2–2.1 mm), with oblong-oval habitus, broadest at elytral middle. *Coloration*: Dorsally piceous, with dark brown anterior part of head and sides of pronotum; head appendages and legs yellowish red, metathoracic legs darker distally (Fig. 5).

*Surface sculpture*: Head with sparse punctation (spaces between punctures 2–3 times size of punctures), evidently finer and sparser anteriorly; diameter of punctures smaller than to almost equal to diameter of cells of microreticulation. Pronotum with much sparser and finer punctation than head. Elytra with extremely sparse and fine punctation, inconspicuous. Pronotum and elytra with slightly impressed microreticulation, dorsal surface shiny. Head with microreticulation stronger. Metaventrite and metacoxae distinctly microreticulate, metacoxal plates with longitudinal strioles and transverse wrinkles. Abdominal ventrites with distinct microreticulation, strioles, and very fine and sparse punctation.

*Structures*: Pronotum without lateral bead. Base of prosternum and neck of prosternal process with distinct ridge, smooth and rounded anteriorly. Blade of prosternal process lanceolate, relatively narrow, slightly convex, with distinct lateral bead and few setae; neck and blade of prosternal process evenly jointed. Abdominal ventrite 6 broadly rounded apically.

*Male*: Antenna simple (Fig. 5). Pro- and mesotarsomeres 1–3 not dilated. Protarsomere 4 cylindrical, narrow, with large, thick, strongly curved anterolateral hook-like seta. Protarsomere 5 ventrally with anterior row of 20 and posterior row of five relatively long, not pointed setae (Fig. 13D). Median lobe slightly curved, not tapering to apex in lateral view, apex divided into three small prolongations. Paramere without dorsal notch, with long, rather dense, thin setae, situated along dorsal margin, not clearly divided into subdistal and proximal (Fig. 13A–C). Abdominal ventrite 6 broadly rounded apically, without or with 1–3 fine transverse lateral strioles on each side.

*Holotype*: TL-H 3.7 mm, TL 4 mm, MW 2 mm.

*Female*: Pro- and mesotarsi not modified. Abdominal ventrite 6 without lateral strioles.

###### Distribution.

Papua: Puncak Regency. The species is known only from the type locality (Fig. 19).

Iratoi is a mixed village of both Edofi and Wano people. It is located just beyond the foot hills coming out of Wano land at the northwestern border of their traditional territory. This area is quite low at an elevation of ca. 200 m, which offers many opportunities to easily access the many small streams and puddles which *Exocelina* inhabits.

###### Etymology.

The name refers to Iratoi, the type locality. The name is a noun in the nominative singular standing in apposition.

#### *Exocelina likui*-group

##### 5. Exocelina likui sp. n.

Taxon classificationAnimaliaColeopteraDytiscidae

http://zoobank.org/3B1B6701-679A-4646-BCEF-8F09A5ECBF88

Figs 7 , 15

###### Type locality.

Papua: Puncak Jaya Regency, south from Iratoi, 03°23'12.5"S; 137°14'43.5"E.

###### Type material.

*Holotype*: male “Indonesia: Papua Province, S Iratoi, forest, 220m, 21.v.2015, -3,38095162063837, 137,311441982164, Sumoked & Balke (Pap036)”, “M.Balke 6980” [green text] (MZB). *Paratypes*: 1 male, 2 females with the same label as the holotype, one female with an additional label “M.Balke 6981” [green text] (NHMW, ZSM).

###### Diagnosis.

Beetle small, oblong, dark brown to piceous, dorsal punctation dense and coarse, microreticulation distinctly impressed; pronotum without lateral bead; male antennae simple; male protarsomere 4 cylindrical, narrow, with large, thick, strongly curved anterolateral hook-like seta; median lobe slightly curved, with broadly pointed apex in lateral view and abruptly narrowed apically, with apex truncate in ventral view; paramere without dorsal notch, with subdistal setae very dense, strong, long and proximal setae very sparse, thin, small, weakly visible.

In shape of median lobe, the species resembles *E. takime* (Balke, 1998) but distinctly differs from it in absence of the pronotal bead and strong dorsal sculpture. From *E. pui* sp. n., it differs in dense and coarse dorsal punctation and in having medial lobe apically more pointed in lateral view, without lateral setae.

###### Description.

**Figures 7–10.**
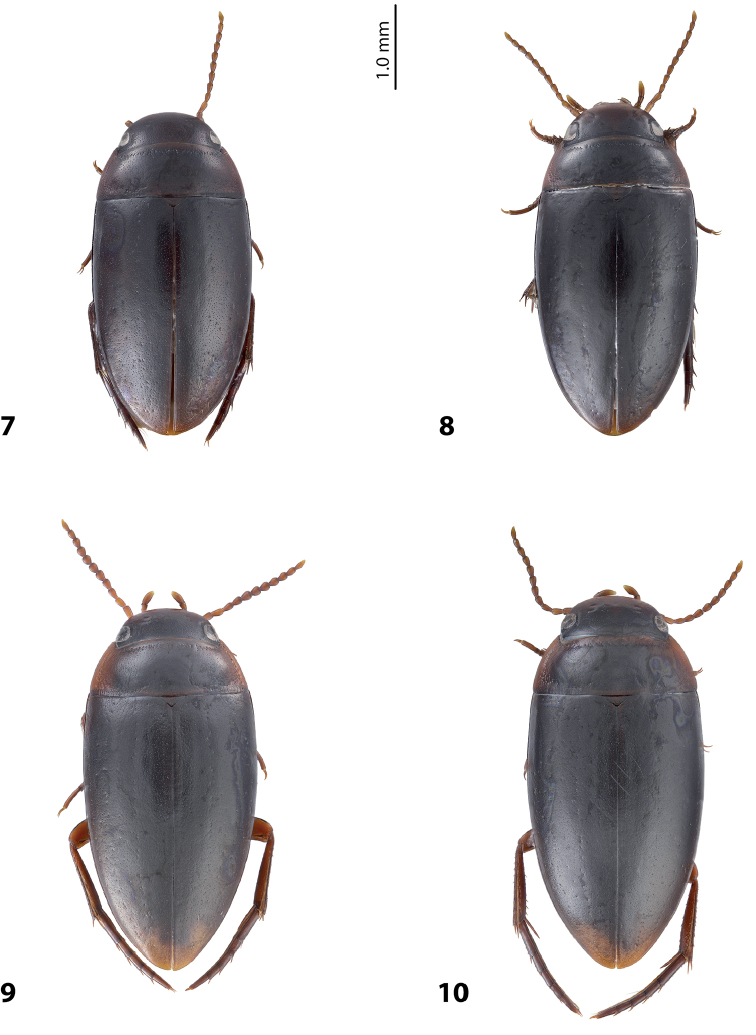
Habitus and coloration **7** *Exocelina likui* sp. n. **8** *E. pui* sp. n. **9** *E. wigodukensis* sp. n. **10** *E. pulukensis* sp. n.

*Size and shape*: Beetle small (TL-H 3.2–3.6 mm, TL 3.8–4.0 mm, MW 1.85–1.95 mm), with oblong habitus, broadest at elytral middle. *Coloration*: Dorsally dark brown to piceous, with head and pronotum paler; head appendages and legs reddish brown distally (Fig. 7).

*Surface sculpture*: Head with dense, coarse punctation (spaces between punctures 1–2 times size of punctures), finer anteriorly and posteriorly; diameter of punctures equal to diameter of cells of microreticulation. Pronotum with slightly sparser punctation than head. Elytra with dense, coarse punctation, coarser than on pronotum. Pronotum and elytra with distinctly impressed microreticulation, dorsal surface submatt. Head with microreticulation stronger. Metaventrite and metacoxae distinctly microreticulate, with sparse but distinct punctation, metacoxal plates with longitudinal strioles and transverse wrinkles. Abdominal ventrites with distinct microreticulation, strioles, and sparse, coarse punctation, especially on four last abdominal ventrites.

*Structures*: Pronotum without lateral bead. Base of prosternum and neck of prosternal process with distinct ridge, smooth, broadly rounded anteriorly. Blade of prosternal process lanceolate, relatively narrow, very slightly convex, with distinct lateral bead and few setae; neck and blade of prosternal process evenly jointed. Abdominal ventrite 6 broadly rounded apically.

*Male*: Antenna simple (Fig. 7). Pro- and mesotarsomeres 1–3 not dilated. Protarsomere 4 cylindrical, narrow, with large, thick, strongly curved anterolateral hook-like seta. Protarsomere 5 ventrally with anterior band of more than 40 and posterior row of 14 relatively long, not pointed setae (Fig. 15D). Median lobe slightly curved, with broadly pointed apex in lateral view, and abruptly narrowed apically, with apex slightly truncate in ventral view. Paramere without dorsal notch, with subdistal setae very dense, strong, long and proximal setae very sparse, thin, small, weakly visible. (Fig. 15A–C). Abdominal ventrite 6 broadly rounded apically, 5–6 lateral strioles on each side.

*Holotype*: TL-H 3.5 mm, TL 3.9 mm, MW 1.9 mm.

*Female*: Pro- and mesotarsi not modified. Abdominal ventrite 6 without lateral strioles.

###### Distribution.

Papua: Puncak Jaya Regency. The species is known only from the type locality (Fig. 19).

###### Etymology.

The species is named after Michael Wild’s best Wano friend Liku who grew up at Iratoi and hunted many times in the area where the species was collected. The name is a noun in the genitive case.

#### *Exocelina pui*-group

##### 6. Exocelina pui sp. n.

Taxon classificationAnimaliaColeopteraDytiscidae

http://zoobank.org/19F321EA-88F3-4766-ACF9-9DF953EA40AD

Figs 8 , 16

###### Type locality.

Papua: Puncak Regency, Puluk area, 03°35'56.1"S; 137°27'53.7"E.

###### Type material.

*Holotype*: male “Indonesia: Papua, Wano Land, red clay creek nr cave, 1100m, 3.ix.2014, nr -3.587955 137.5114945, Balke & Wild (Pap024)”, “M.Balke 6518” [green text] (MZB). *Paratypes*: 5 males with the same label as the holotype, one of them with an additional label “M.Balke 6519” [green text] (ZSM).

###### Diagnosis.

Beetle small, dark brown to piceous, with paler anterior part of head and sides of pronotum, dorsal punctation fine, microreticulation distinctly impressed; pronotum without lateral bead; male antennae simple; male protarsomere 4 cylindrical, narrow, with large, thick, strongly curved anterolateral hook-like seta; median lobe slightly curved, with truncate apex in lateral view and abruptly narrowed apically, with apex concave in ventral view, having short, thick subdistal setae laterally; paramere without dorsal notch, with subdistal setae dense, strong, long and proximal setae very sparse, thin, small, weakly visible.

In habitus shape, coloration, and absence of the pronotal bead, *E. pui* sp. n. strongly resembles the small species from the *E. ekari*-group (one of them is its co-occurring species) but it distinctly differs from them in having different shape of the median lobe and paramere. The shape of the median lobe is very similar to that of *E. rivulus* (Balke, 1998), also a co-occurring species, from which *E. pui* sp. n. can be easily distinguished in its smaller size and absence of the pronotal bead. From *E. likui* sp. n., it differs in distinctly finer dorsal punctation and in having median lobe truncate in lateral view, with lateral setae.

###### Description.

*Size and shape*: Beetle small (TL-H 3.35–3.6 mm, TL 3.95–4.0 mm, MW 1.95–2.0 mm), with oblong habitus, broadest at elytral middle. *Coloration*: Head dark brown, almost piceous posteriorly and reddish brown anteriorly. Pronotum and elytra dark brown to piceous, pronotal sides reddish brown; head appendages and legs reddish brown (Fig. 8).

*Surface sculpture*: Head with fine and relatively dense punctation (spaces between punctures 1–3 times size of punctures), finer and sparser anteriorly; diameter of punctures almost equal to diameter of cells of microreticulation. Pronotum with sparser and finer punctation than head. Elytra with very sparse and fine punctation, often inconspicuous. Pronotum and elytra with distinctly impressed microreticulation, dorsal surface less shiny. Head with microreticulation stronger. Metaventrite and metacoxae distinctly microreticulate, metacoxal plates with longitudinal strioles and transverse wrinkles. Abdominal ventrites with distinct microreticulation, strioles, and very fine and sparse punctation.

*Structures*: Pronotum without lateral bead. Base of prosternum and neck of prosternal process with distinct ridge, smooth, broadly rounded anteriorly. Blade of prosternal process lanceolate, relatively narrow, very slightly convex, with distinct lateral bead and few setae; neck and blade of prosternal process evenly jointed. Abdominal ventrite 6 broadly rounded apically.

*Male*: Antenna simple (Fig. 8). Pro- and mesotarsomeres 1–3 not dilated. Protarsomere 4 cylindrical, narrow, with large, thick, strongly curved anterolateral hook-like seta. Protarsomere 5 slightly concave ventrally, with anterior band of more than 40 and posterior row of 15 relatively long, not pointed setae (Fig. 16D). Median lobe slightly curved, with truncate apex in lateral view and abruptly narrowed apically, with apex concave in ventral view, having short, thick subdistal setae laterally. Paramere without dorsal notch, with subdistal setae dense, strong, long and proximal setae very sparse, thin, small, weakly visible (Fig. 16A–C). Abdominal ventrite 6 broadly rounded apically, with 6–8 fine transverse lateral strioles on each side.

*Holotype*: TL-H 3.6 mm, TL 4.0 mm, MW 2.0 mm.

*Female*: Pro- and mesotarsi not modified. Abdominal ventrite 6 without lateral strioles.

###### Distribution.

Papua: Puncak Regency. The species is known only from the type locality (Fig. 19).

###### Etymology.

The species is named after a young Wano man Pu, who grew up in Puluk and accompanied his father (now deceased) on many hunting trips in the area where this species was collected. The name is a noun in the genitive case.

#### *Exocelina ransikiensis*-group

##### 7. Exocelina ransikiensis

Taxon classificationAnimaliaColeopteraDytiscidae

Shaverdo, Panjaitan & Balke, 2016

Exocelina undescribed sp. MB1269: Toussaint et al. 2014: Supplementary figs 1–4, Tab. 2.
Exocelina ransikiensis Shaverdo, Panjaitan & Balke, 2016d: 104, figs 1–6.

###### Type locality.

West Papua: Manokwari Regency, approximately 10 km NW from Ransiki, Kali Way, 01°25'03"S; 134°01'49"E.

###### Type material studied.

*Holotype*: male “West Papua, ca. 10 km NW Ransiki, Kali Way, 1300 m, 01°25'03"S, 134°01'49"E, 03.III.2007”, “HolotypUS *Exocelina ransikiensis* sp. n. des. H. Shaverdo, R. Panjaitan & M. Balke, 2016” [red] (MZB). *Paratypes*: 6 males, 2 females with the same label as the holotype and additionally with red labels “PARAtypUS *Exocelina ransikiensis* sp. n. des. H. Shaverdo, R. Panjaitan & M. Balke, 2016” (CASk, NHMW, ZSM). 1 male “West Papua, old road Ransiki to Anggi, 1160 m, 01°25'53.6"S, 134°02'45.6"E, Balke (BH 03)”, “M.Balke 1269” [green] (ZSM).

###### Additional material.

**West Papua**: 3 males “Indonesia: Papua Barat, Manokwari to Kebar, forest stream, 302m, 3.xi.2013, -0.80058566 133.33216397, Balke (BH023)”, one male with an additional label “M.Balke 6185” [green text] (NHMW, ZSM). 4 males, 2 females “Indonesia: Papua Barat, Kebar to Aibogar, forest stream, 644m, 4.xi.2013, -0.85339769 132.87133633, Balke (BH024)”, one male with an additional label “M.Balke 6188” [green text] (NHMW, ZSM). 1 male “Indonesia: Papua Barat, Fumato, forest stream, 820m, 5.xi.2013, -0.90427148 132.71981431, Balke (BH027)” (ZSM). 1 male “Indonesia: Papua Barat, Tamrau Mts N of Kebar, forest stream, 750m, 7.xi.2013, -0.783199 133.072143, Balke (BH033)” (ZSM). 4 males “Indonesia: Papua Barat, Tamrau Mts N of Kebar, forest stream, puddles, 1050m, 7.xi.2013, -0.774519 133.069929, Balke (BH034)” (NHMW, ZSM). **Papua**: 1 female “Indonesia: Papua, Road Nabire-Enarotali KM 108, 140m, 23.x.2011, 03 30.258S 135 54.840E, Balke (PAP16)”, “M.Balke 7235” [green text] (ZSM). 1 female “Irian Jaya: Nabire distr., road Nabire-Ilaga, km 54, 03°29'517"S 135°43'913"E, 750m, iv.1998 M. Balke leg.” (NHMW).

###### Diagnosis.

Small, with oblong with subparallel sides to broadly oval habitus; coloration red to dark brown; dorsal surface with strong punctation and microreticulation, matt; pronotum with distinct lateral bead; male antennomeres simple; male protarsomere 4 with large, thick, strongly curved anterolateral hook-like seta; median lobe slightly tapering in ventral view and with curved apex in lateral view; paramere without notch on dorsal side, with thin, sparse, inconspicuous setae. For the complete description, please see Shaverdo et al. (2016d).

###### Notes on morphological variability.

Recently discovered beetles from the western part of Manokwari Regency show distinct differences in the size, body shape, and coloration from those of the type series. They are larger: TL-H 3.25–3.7 mm (representatives of the type series: TL-H 2.85–3.2 mm) and have darker coloration: dark brown, with paler anterior margin of the head and sides of the pronotum. Very interesting is the variability of the body shape: the beetles from Ransiki and Nabire are oblong, with subparallel sides, the beetles from the Tamrau Mountains (localities BH023, BH033, and BH034) also have this body shape but they are distinctly larger, whereas the beetles from Kebar-Aibogar have a more rounded habitus, which is distinctly broadly oval in the specimen from Fumato. That represents almost gradual change of the body shape from oblong, parallel-sided in the east to broadly oval in the west. No variability has been found in the shape of the median lobe and paramere, or in the surface sculpture. Therefore, at present, we treat all new material as *E. ransikiensis* bearing in mind that more material is needed for a final conclusion.

###### Distribution.

West Papua: Manokwari Regency and Papua: Nabire Regency (Fig. 19).

#### *Exocelina wigodukensis*-group

This group includes two species, which have one unique character: few (usually three) last subdistal setae of the paramere, standing isolated, are modified having indistinct or very evident basal prolongation (Figs 17C, 18C). Such a modification is also characteristic for some subdistal setae, which are in the “brash” (see in the description below), but it is less evident.

##### 8. Exocelina wigodukensis sp. n.

Taxon classificationAnimaliaColeopteraDytiscidae

http://zoobank.org/8D1B2AFF-4961-480C-BEC2-80E886CB7C78

Figs 9 , 17

###### Type locality.

Papua: Puncak Jaya Regency, Wigoduk, 03°38'14.52"S; 137°46'57.78"E.

**Type**

###### material.

*Holotype*: male “Indonesia: Papua, Wigoduk, 1800m, 29.xi.2014, S3°38'14.52", E137°46'57.78", M. Wild (W19)” (MZB). *Paratypes*: 6 males, 3 females with the same label as the holotype, two males with the additional labels “M.Balke 6528” [green text] and “M.Balke 6529” [green text] (NHMW, ZSM). 4 males “Indonesia: Papua, Mokndoma, 2150m, 5.ix.2014, S3°38'38.94", E137°46'30", M. Wild” (NHMW, ZSM).

###### Additional material.

3 males, 10 females “Indonesia: Papua, Mokndoma, 2150m, 5.ix.2014, S3° 38’ 38.94” E137° 46’ 30”, M. Wild”, one female additionally with label “M.Balke 6530” [green text] (ZSM).

###### Diagnosis.

Beetle medium-sized, piceous, with pronotum paler anteriorly and laterally, dorsal punctation dense and coarse, microreticulation strongly impressed; pronotum without lateral bead; male antennae evidently modified: antennomeres 3–4 and 9–10 stout, antennomeres 5–8 distinctly enlarged, larger than other antennomeres; protarsomere 4 with large, thick, strongly curved anterolateral hook-like seta; protarsomere 5 slightly concave ventrally; median lobe slightly curved, narrow, with apex slightly curved downwards as very small “beak” in lateral view; with slightly concave apex and subparallel sides in ventral view; paramere without dorsal notch, subdistal setae numerous, long, dense, strong, proximal setae thin and sparse, inconspicuous.

The species is similar to *E. pulukensis* sp. n. but distinctly differs from it in having more strongly modified male antennae, thinner and stronger curved apex of the median lobe in lateral view, and three isolated setae of the paramere with very small basal prolongations. Also see under *E. pulukensis* sp. n.

###### Description.

*Size and shape*: Beetle medium-sized (TL-H 3.7–4.35 mm, TL 4.05–4.7 mm, MW 1.95–2.3 mm), with oblong-oval habitus, broadest at elytral middle. *Coloration*: Head piceous, with narrow reddish brown anterior margin. Pronotum piceous on disc and posterior part and reddish brown anteriorly and laterally. Elytra uniformly piceous. Head appendages reddish brown to dark brown. Legs yellowish red proximally and reddish brown distally (Fig. 9). Ventrum piceous, paler on abdominal ventrites; prosternum, epipleurae, abdominal ventrite 1, and apical part of abdominal ventrite 6 reddish brown. Coloration of teneral specimens paler.

*Surface sculpture*: Head with dense punctation (spaces between punctures 1–2 times size of punctures), evidently finer and sparser anteriorly; diameter of punctures almost equal to diameter of cells of microreticulation. Pronotum with sparser and finer punctation than head. Elytra with coarse and dense punctation, coarser than on pronotum. Pronotum and elytra with strongly impressed microreticulation, dorsal surface matt. Head with microreticulation stronger. Metaventrite and metacoxae distinctly microreticulate and punctate, metacoxal plates with longitudinal strioles and transverse wrinkles. Abdominal ventrites with distinct microreticulation, strioles, and distinct sparse punctation.

*Structures*: Pronotum without lateral bead. Base of prosternum and neck of prosternal process with distinct ridge, slightly rounded anteriorly, with small transverse wrinkles. Blade of prosternal process lanceolate, relatively broad, with distinct lateral bead, with small transverse wrinkles on both sides along lateral bead and convex and smooth middle; neck and blade of prosternal process evenly jointed. Abdominal ventrite 6 broadly rounded or slightly truncate apically.

*Male*: Antenna modified (Fig. 9); antennomeres 3–4 and 9–10 stout, antennomeres 5–8 distinctly enlarged, larger than other antennomeres. Pro- and mesotarsomeres 1–3 slightly dilated. Protarsomere 4 not modified, with large, thick, strongly curved anterolateral hook-like seta. Protarsomere 5 slightly concave ventrally, with anterior band of more than 30 and posterior row of 7 relatively long, not pointed setae (Fig. 17D). Median lobe slightly curved, narrow, with apex slightly curved downwards as very small “beak” in lateral view; with slightly concave apex and subparallel sides in ventral view. Paramere without dorsal notch, subdistal setae numerous, long, dense (forming “brash”), strong, the three most proximal of them standing isolated and slightly modified having small basal prolongations; proximal setae thin and sparse, inconspicuous (Fig. 17A–C). Abdominal ventrite 6 with 8–12 lateral strioles on each side.

*Holotype*: TL-H 4.45 mm, TL 4.15 mm, MW 2.2 mm.

*Female*: Antennomeres stout but thinner than in male. Pro- and mesotarsi not modified. Abdominal ventrite 6 without lateral strioles.

###### Variability.

There are three specimens (see “Additional material”) of much smaller size (TL-H 3.8–3.95 mm, TL 3.2–3.6 mm, MW 1.85–1.9 mm; for *E. wigodukensis* sp. n. from Mokndoma: TL-H 4.05–4.35 mm, TL 4.3–4.7 mm, MW 2.1–2.3 mm) and with slightly different shape of the median lobe (less curved downwards apex) among the beetles of the population from Mokndoma. More material from the region is necessary to conclude whether two distinct but very similar species occur here or it is just a matter of variability.

###### Distribution and habitat.

Papua: Puncak Jaya Regency. The species is known from two localities: Wigoduk and Mokndoma (Fig. 19).

At 2150 m, Mokndoma is an area of high cloud forest. Although this area lies within the territory of the Wano tribe, the word Mokndoma is from the neighboring Dem language. Mok means “flat” and Ndoma means “ground”. So the name of the place is literally “Flat ground”. This is the current home of the second author. In many places at Mokndoma, the ground is boggy with lots of moss and tannin stained streams and ponds. It is an almost mystical place featuring moss tunnels, bog grass, mountain rhododendrons, woody epiphytes, wild ginger and many shrubby small coniferous trees. Upon leaving the open flat area and heading into the thick jungle to the east, west or south, one finds many small streams (Fig. 1) and puddles in which *Exocelina* beetles abound.

Wigoduk is the name of a valley system to the northeast of Mokndoma. It is about onehour hike from the second author’s house site in Mokndoma. Wigoduk is located at 1800 m, right on the eastern bank of a wide bend in the Nggoduk River (Fig. 2). The Nggoduk River is approximately 6–8 m wide and most times only knee deep. In the Wigoduk valley, it meanders along, but upon exiting the valley, turns turbulent as it hurries down the mountain to the larger Yamo River at the bottom of a large east to west running valley at about 1000 m elevation. The Nggoduk River banks are made of smooth rocks and pebbles, a superb site for collecting many different Coleoptera. *Exocelina* beetles are found around this area and on the trail from Mokndoma to Wigoduk in many very small streams and puddles.

###### Etymology.

The name refers to Wigoduk, the type locality. The name is an adjective in the nominative singular.

##### 9. Exocelina pulukensis sp. n.

Taxon classificationAnimaliaColeopteraDytiscidae

http://zoobank.org/ABE7DB85-B0C4-4DE3-9C58-42C1CC8EFB78

Figs 10 , 18

###### Type locality.

Papua: Puncak Jaya Regency, Puluk area, 03°37'28.5"S; 137°28'35.8"E.

###### Type material.

*Holotype*: male “Indonesia: Papua, Wano Land, S of pass to lake plains, 1700m, 2.ix.2014, -3,6117913 137,5277983, Balke & Wild (Pap022)” (MZB). *Paratypes*: 13 males, 3 females with the same label as the holotype, one male with an additional label “M.Balke 6514” [green text] (NHMW, ZSM). 6 males, 4 female “Indonesia: Papua, Wano Land, N of pass to lake plains, 2000m, 2.ix.2014, -3,6117913 137,5277983, Balke & Wild (Pap023)”, one male with an additional label “M.Balke 6509” [green text] (NHMW, ZSM).

###### Additional material.

3 males, 1 female “Indonesia: Papua, Wano Land, Puluk, 1320m, 1.ix.2014, -3.660272 137.5207436, Balke & Wild (Pap020)”, one male with an additional label “M.Balke 6521” [green text] (ZSM). 1 male “Indonesia: Papua, Wano Land, red clay creek nr cave, 1100m, 3.ix.2014, nr -3.587955 137.5114945, Balke & Wild (Pap024)”, “M.Balke 6515” [green text] (ZSM).

###### Diagnosis.

Beetle medium-sized, piceous, with pronotum paler anteriorly and laterally, dorsal punctation dense and coarse, microreticulation strongly impressed; pronotum without lateral bead; male antennomeres 3–10 stout; protarsomere 4 with large, thick, strongly curved anterolateral hook-like seta; protarsomere 5 slightly concave ventrally; median lobe slightly curved, with apex rounded, slightly curved downwards in lateral view, in ventral view narrowed subdistally, with subparallel sides and slightly asymmetrical, concave apex; paramere without dorsal notch, subdistal setae numerous, long, dense, strong, three isolated setae of the paramere with distinct basal prolongations, proximal setae thin and sparse, inconspicuous.

The species is similar to *E. wigodukensis* sp. n. but differs from it in having less modified antennae, different shape of the median lobe (broader and without apical “beak” in lateral view and narrowed subdistally and with slightly asymmetrical apex in ventral view), and in having three isolated setae of the paramere with distinct basal prolongations.

###### Description.

*Size and shape*: Beetle medium-sized (TL-H 3.7–4.25 mm, TL 4.1–4.6 mm, MW 2–2.25 mm), with oblong-oval habitus, broadest at elytral middle. *Coloration*: as in *E. wigodukensis* sp. n. (Fig. 10).

*Surface sculpture*: as in *E. wigodukensis* sp. n.

*Structures*: as in *E. wigodukensis* sp. n. Abdominal ventrite 6 broadly rounded apically.

*Male*: Antenna only slightly modified (Fig. 10): antennomeres 3–10 stout. Pro- and mesotarsomeres 1–3 slightly dilated. Protarsomere 4 not modified, with large, thick, strongly curved anterolateral hook-like seta. Protarsomere 5 slightly concave ventrally, with anterior band of ca. 30 and posterior row of six relatively long, not pointed setae (Fig. 18D). Median lobe slightly curved, with apex rounded, slightly curved downwards in lateral view; in ventral view, narrowed subdistally, with subparallel sides and slightly asymmetrical, concave apex. Paramere without dorsal notch, subdistal setae numerous, long, dense, strong, the three most proximal of them standing isolated and strongly modified having distinct basal prolongations; proximal setae thin and sparse, inconspicuous (Fig. 18A–C). Abdominal ventrite 6 with 4–7 lateral strioles on each side.

*Holotype*: TL-H 4.2 mm, TL 4.6 mm, MW 2.2 mm.

*Female*: Antennae distinctly more slender than in males. Pro- and mesotarsi not modified. Abdominal ventrite 6 without lateral strioles.

###### Variability.

The males from the localities Pap020 and Pap024 (see “Additional material”) have thicker and shorter median lobe, with its apex distinctly broader in lateral view and more concave in ventral view. More material from the region is necessary to conclude whether two distinct but very similar species occur here or it is just a matter of variability.

###### Distribution.

Papua: Puncak Jaya Regency. The species is known from Puluk area (Fig. 19).

At Puluk (1370 m), there are three permanent Wano families living, and near their houses, the small trees and bushes are kept trimmed back. The soil is dark, and very fertile. Off into the jungle, around their houses in a circumference of approximately 30–50 m, secondary growth is always encroaching on the hamlet site. Outwards to 250 m beyond the secondary growth, the jungle is lush, but somewhat thinned out, since they clear out smaller trees, and underbrush for firewood and materials for building houses and gardens. Their gardens are out beyond that, and are roughly made and maintained. Beyond that, the jungle is pristine. Just in the jungle to the west of the hamlet site is a small stream where *Exocelina* abound (the villagers nearest drinking and bathing source). To the north of the hamlet in the mountains, there are many smaller streams and puddles coming teeming with beetles.

###### Etymology.

The name refers to Puluk area where the species were found. The name is an adjective in the nominative singular.

## Key to species

The key is based mostly on the male characters. In many cases females cannot be assigned to species due to similarity of their external and internal structures (for female genitalia see figs 17a and 17b in Shaverdo et al. (2005)). Some species are rather similar in point of external morphology, therefore, in most cases the male genitalia need to be studied for reliable species identification. Numbers in brackets refer to an arrangement of the species descriptions above.

**Table:** 

1	Pronotum with lateral bead	**2**
–	Pronotum without lateral bead	**3**
2	Beetle larger, TL-H 5.3–5.8 mm (Fig. 3). Medial lobe with lateral setae (Fig. 11B). Paramere robust, with notch on dorsal side and very dense, strong setae on subdistal part (Fig. 11C)	(1) ***ascendens***
–	Beetle smaller, TL-H 2.85–3.2 mm (fig. 1 in Shaverdo et al. 2016d). Medial lobe without lateral setae (fig. 5 in Shaverdo et al. 2016d). Paramere slender, without notch on dorsal side, setae thinner (fig. 6 in Shaverdo et al. 2016d)	(7) ***ransikiensis***
3	Male antennae extremely modified: antennomeres 4-6 excessively large, 3 and 7 strongly enlarged (Fig. 6)	(3) ***bagus***
–	Male antennae simple or differently modified	**4**
4	Apex of median lobe with three small prolongations (Fig. 14A–B). Beetle small, TL-H 3.7–3.9 mm, dorsal punctation inconspicuous, microreticulation weakly impressed (Fig. 5)	(4) ***iratoi* sp. n.**
–	Median lobe with simple apex, other characters variable	**5**
5	Beetle larger, TL-H 3.7–4.75 mm	**6**
–	Beetle smaller, TL-H 3.2–3.6 mm	**8**
6	Beetle shiny, with fine dorsal microreticulation and punctation almost invisible (Fig. 4). Male antennae not modified. Medial lobe with apex narrowly pointed in lateral view (Fig. 12B)	(2) ***tomhansi* sp. n.**
–	Beetle matt, with strong dorsal microreticulation and punctation. Male antennae modified: antennomeres 3–10 stout or some of them distinctly enlarged. Medial lobe with apex more or less rounded in lateral view	**7**
7	Male antennomeres 3–4 and 9–10 stout, antennomeres 5–8 distinctly enlarged, larger than other antennomeres (Fig. 9). Medial lobe with apex narrower and more curved downwards in lateral view (Fig. 17B). Few last, isolated subdistal setae of paramere slightly modified having indistinct basal prolongations (Fig. 17C)	(8) ***wigodukensis* sp. n.**
–	Male antennomeres 3–10 stout (Fig. 10). Medial lobe with apex broader and less curved downwards in lateral view (Fig. 18B). Few last, isolated subdistal setae of paramere strongly modified having distinct basal prolongations (Fig. 18C)	(9) ***pulukensis* sp. n.**
8	Dorsal punctation dense, coarse (Fig. 7). Medial lobe apically more pointed in lateral view, without lateral setae (Fig. 15B)	(5) ***likui* sp. n.**
–	Dorsal punctation very fine (Fig. 8). Medial lobe apically truncate in lateral view, with lateral setae (Fig. 16B)	(6) ***pui* sp. n.**

**Figure 11.**
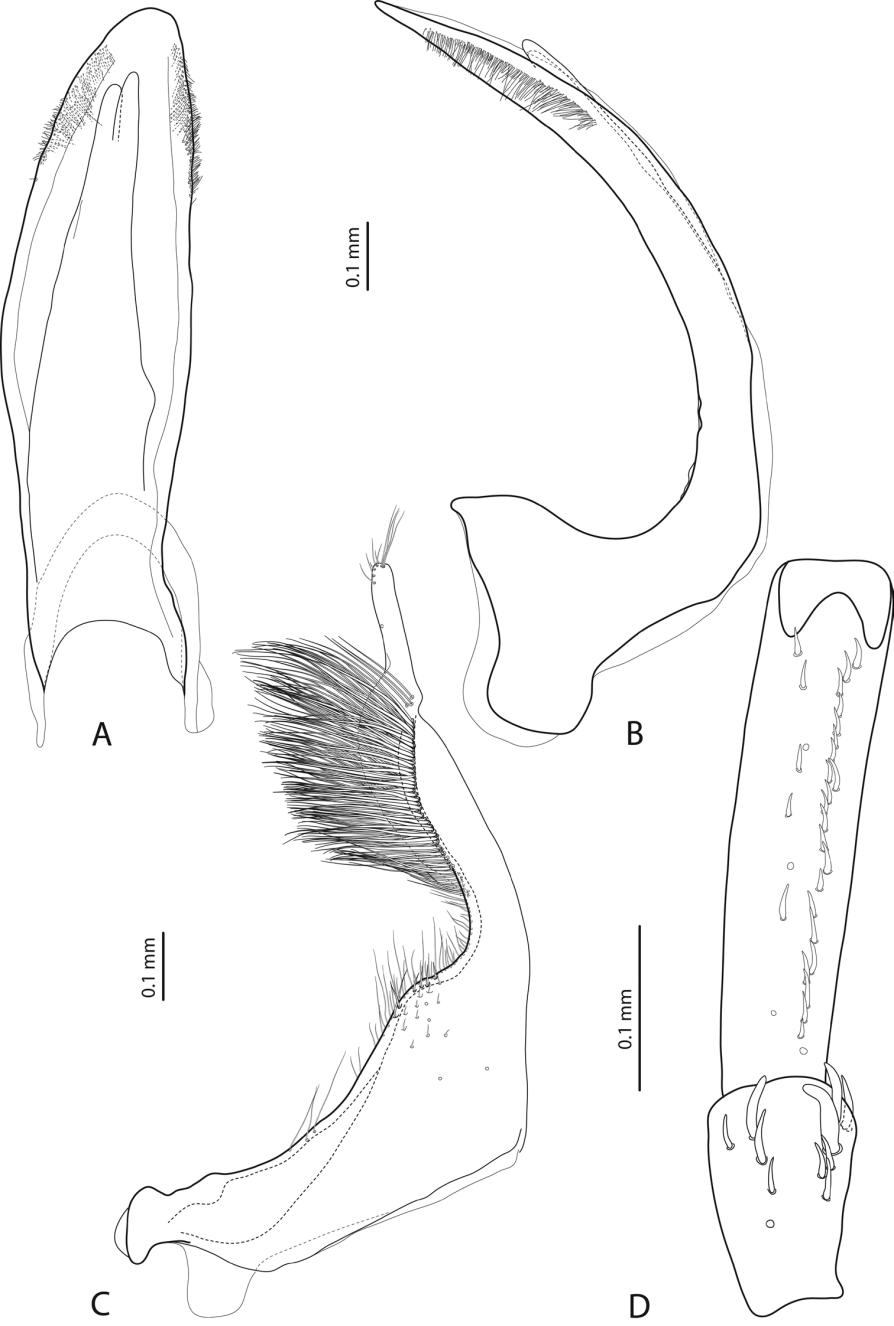
*Exocelina ascendens* (Balke, 1998) **A** median lobe in ventral view **B** median lobe in lateral view **C** paramere in external view **D** male protarsomeres 4–5 in ventral view.

**Figure 12.**
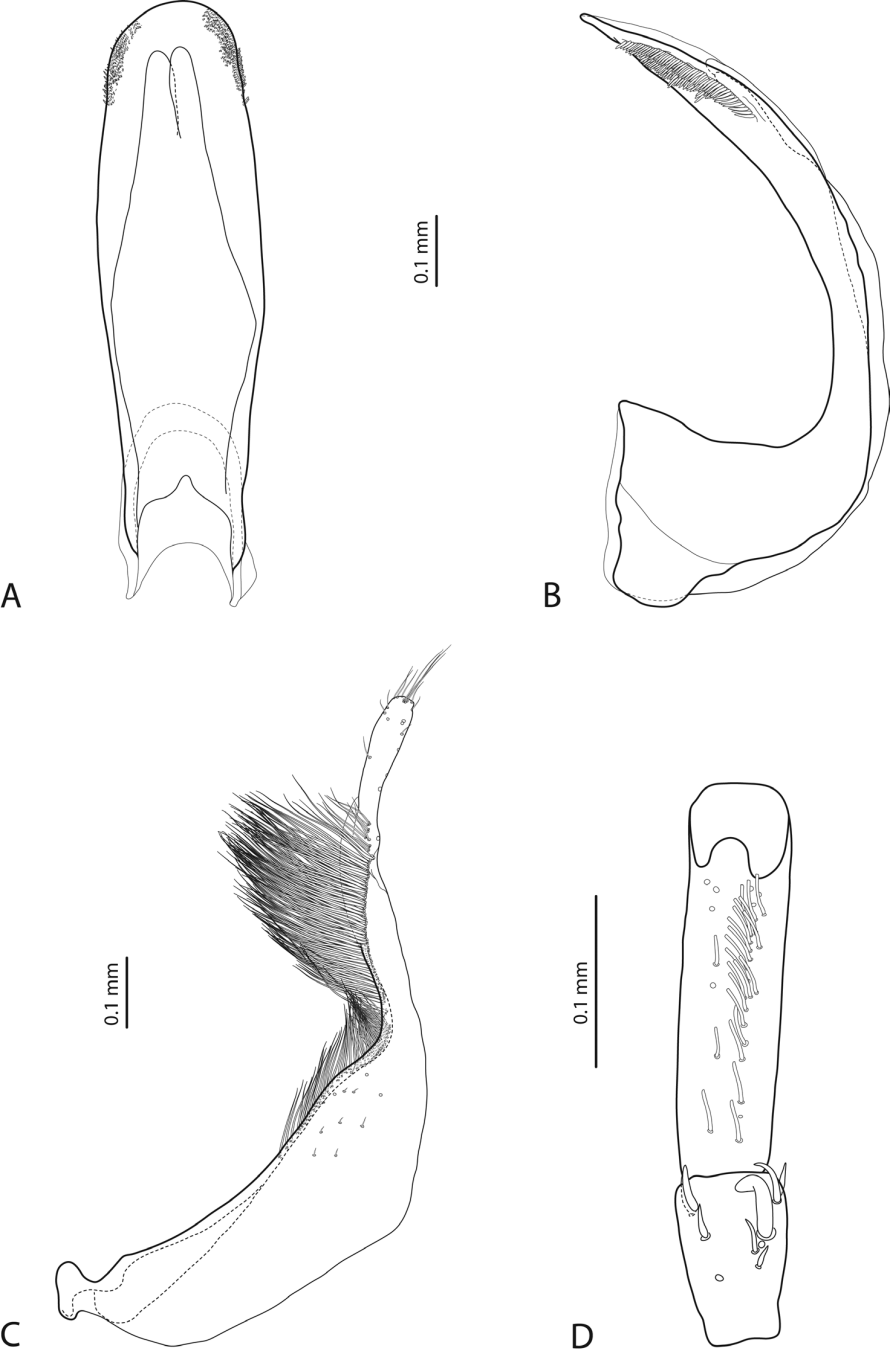
*Exocelina tomhansi* sp. n. **A** median lobe in ventral view **B** median lobe in lateral view **C** paramere in external view **D** male protarsomeres 4–5 in ventral view.

**Figures 13–14.**
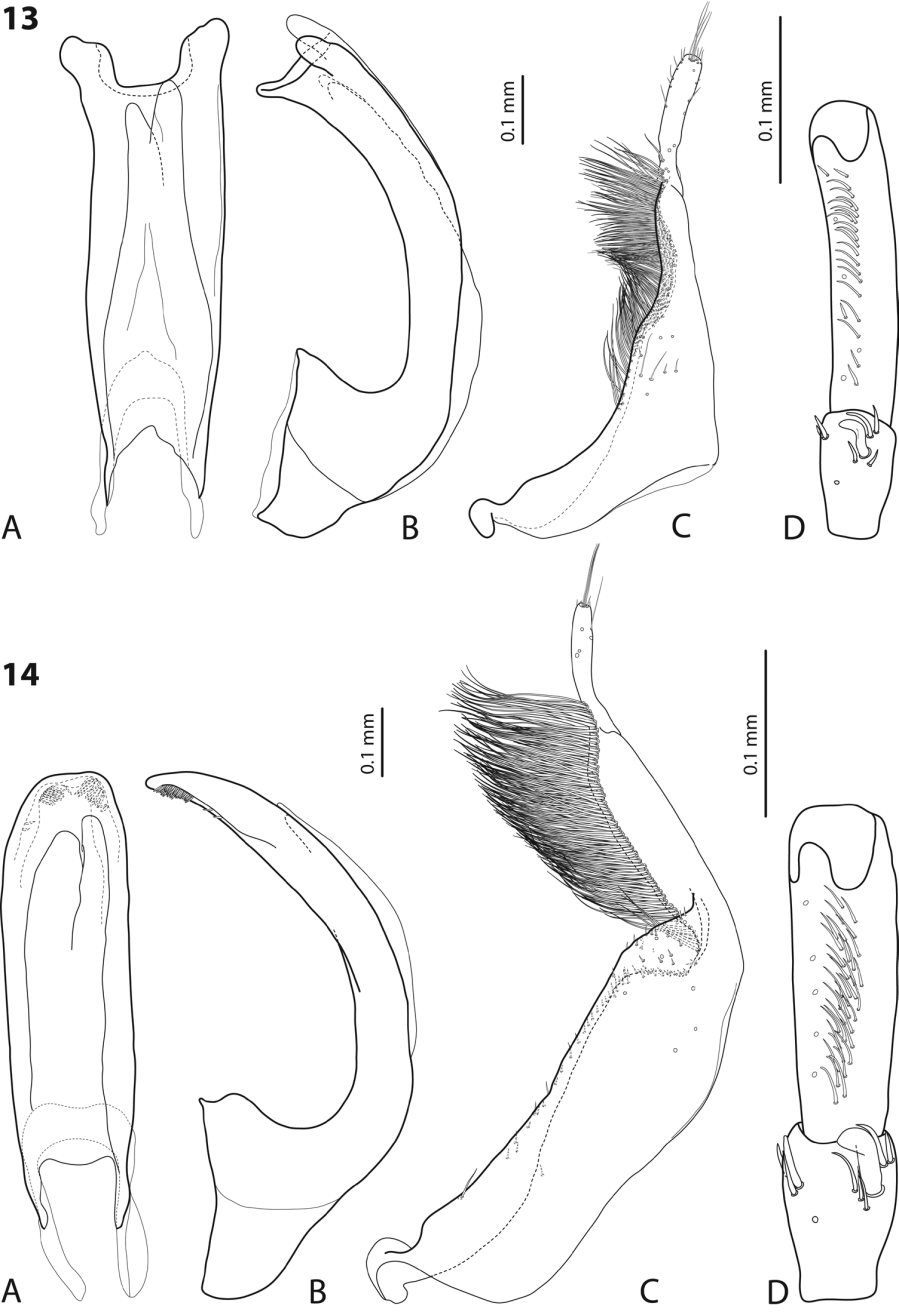
**13** *Exocelina iratoi* sp. n. **14** *E. bagus* (Balke & Hendrich, 2001) **A** median lobe in ventral view **B** median lobe in lateral view **C** paramere in external view **D** male protarsomeres 4–5 in ventral view.

**Figures 15–16.**
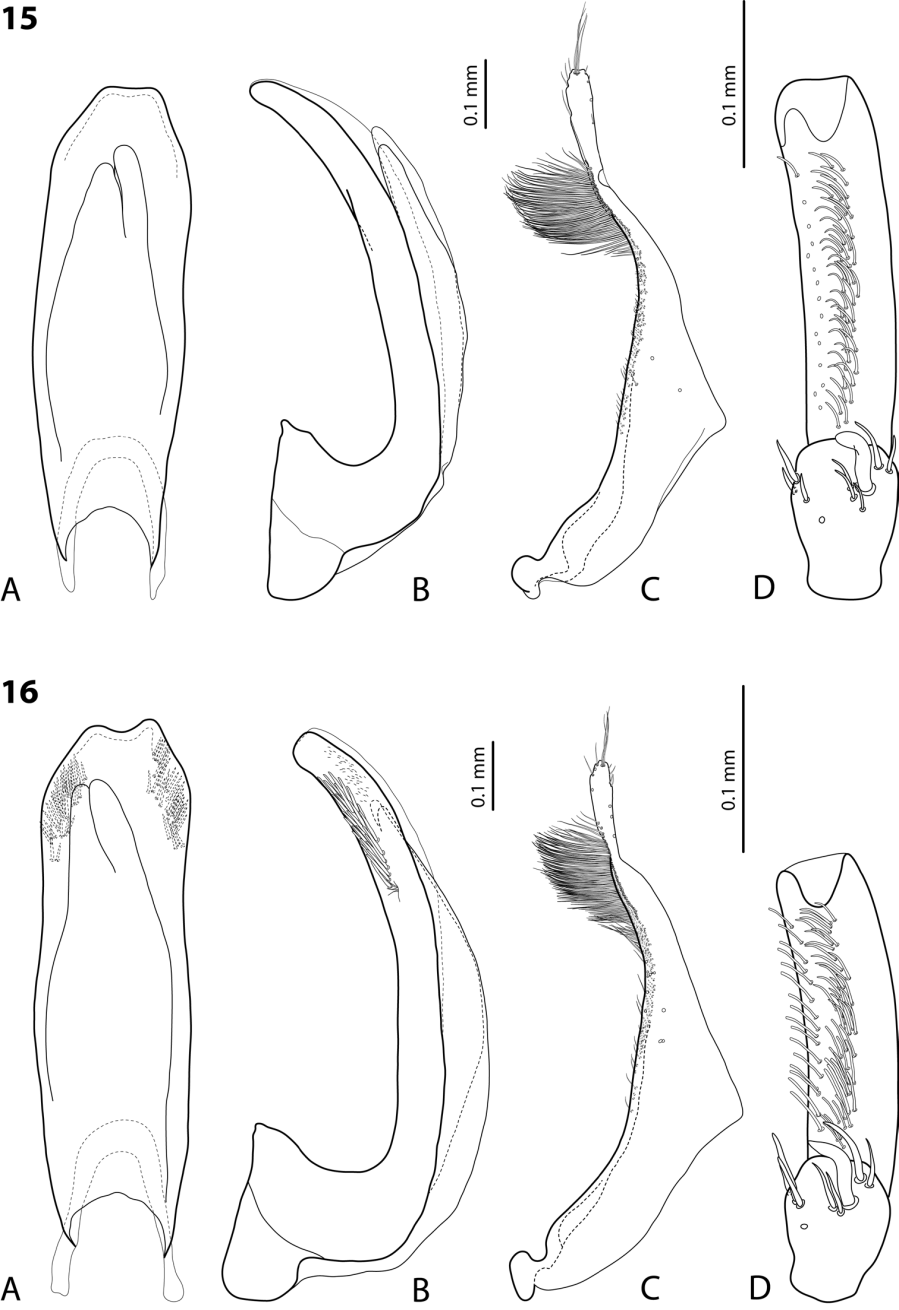
**15** *Exocelina likui* sp. n. **16** *E. pui* sp. n. **A** median lobe in ventral view **B** median lobe in lateral view **C** paramere in external view **D** male protarsomeres 4–5 in ventral view.

**Figures 17–18.**
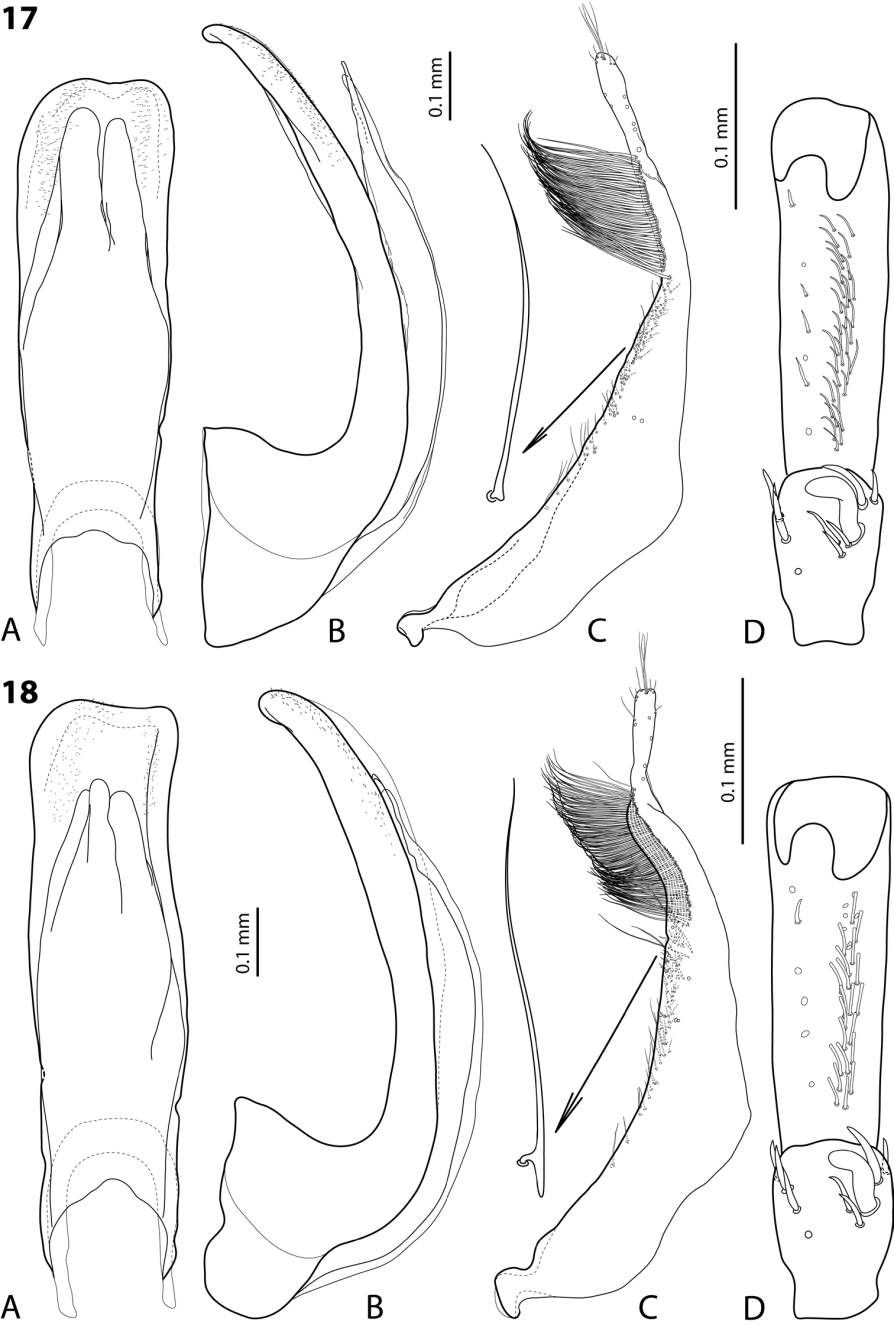
**17** *Exocelina wigodukensis* sp. n. **18** *E. pulukensis* sp. n. **A** median lobe in ventral view **B** median lobe in lateral view **C** paramere in external view **D** male protarsomeres 4–5 in ventral view.

**Figure 19.**
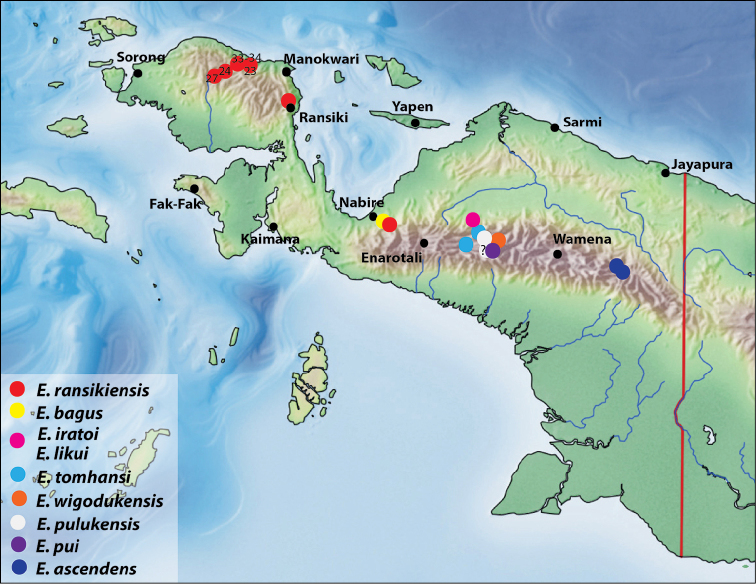
Map of the western part of New Guinea showing the species distributions.

## Supplementary Material

XML Treatment for Exocelina ascendens

XML Treatment for Exocelina tomhansi

XML Treatment for Exocelina bagus

XML Treatment for Exocelina iratoi

XML Treatment for Exocelina likui

XML Treatment for Exocelina pui

XML Treatment for Exocelina ransikiensis

XML Treatment for Exocelina wigodukensis

XML Treatment for Exocelina pulukensis
